# Hispidin Inhibits Ferroptosis Induced by High Glucose via the miR-15b-5p/GLS2 Axis in Pancreatic Beta Cells

**DOI:** 10.1155/2023/9428241

**Published:** 2023-02-21

**Authors:** Fang Wu, Chenxin Shang, Ting Jin, Linhui Shi

**Affiliations:** ^1^Department of Endocrinology, The Affiliated Sir Run Run Shaw Hospital, College of Medicine, Zhejiang University, Hangzhou 310016, Zhejiang, China; ^2^Critical Care Unit, Ningbo Medical Center, Lihuili Hospital, Ningbo University, Ningbo, China

## Abstract

Type 2 diabetes mellitus (T2DM) is a global health issue that lacks effective treatments. Dysfunction and/or death of pancreatic *β*-cells (PBCs) are considered a major cause of T2DM. Therefore, elucidating the mechanisms underlying the death of PBCs might be helpful to develop novel strategies to treat T2DM. Ferroptosis is a newly identified form of cell death that has distinct features. However, knowledge regarding the role of ferroptosis in the death of PBCs remains limited. In the current study, we used high glucose (10 mM) (HG) levels to induce ferroptosis in PBC. We also observed that hispidin, a polyphenol compound that can be isolated from *Phellinus linteus*, could attenuate ferroptosis induced by HG in PBCs. Mechanistic investigations showed that hispidin led to the upregulation of miR-15b-5p, which directly inhibits the expression of glutaminase (GLS2) which plays an essential role in the glutamine metabolism. In addition, we found that overexpression of GLS2 could abrogate the protective effect of hispidin against ferroptosis caused by HG in PBCs. Therefore, our study provides novel insights into the mechanisms that regulate the death of PBCs.

## 1. Introduction

Diabetes mellitus (DM) is a global health issue that affected over 463 million people in 2019 worldwide [1]. Type 2 diabetes mellitus (T2DM) is the major subtype and accounts for approximately 90% of the total cases of DM worldwide [1]. Impaired insulin secretion is considered the core defect in severe T2DM that occurs due to decompensation and dysfunction of pancreatic *β*-cells (PBCs) [2]. Death of PBCs resulting in changes in their mass and function has been recognized as a milestone event, which leads to rapid deterioration of various complications during the progression of T2DM [3]. However, the molecular mechanisms underlying the death of PBCs are not comprehensively understood. Therefore, unveiling the mechanisms and developing effective intervention strategies are necessary.

Ferroptosis is a novel form of cell death that is biochemically, genetically, and morphologically different from other types of cell death such as apoptosis, necrosis, and autophagy [4]. Ferroptosis is associated with intracellular iron overload and iron-dependent accumulation of reactive oxygen species (ROS) [5]. Interestingly, an epidemiological analysis revealed a correlation between T2DM and iron levels in the body [6]. In addition, ROS is considered as an important regulator of T2DM [7]. These findings indicate that ferroptosis may play an essential role in the death of PBCs and the development of T2DM. However, there is limited knowledge regarding the role of ferroptosis in the death of PBCs.

MicroRNAs (miRNAs) are a group of small noncoding RNAs that can bind to the 3′-untranslated region (3′ UTR) of targeted genes and negatively regulate their expression [8]. Numerous miRNAs have been found to affect the death of PBCs. For example, miR-21 can inhibit the death of PBCs via negative regulation of programmed cell death 4 (PDCD4) [9]. MiR-200 regulates the death of PBCs and induces the development of T2DM in vivo [10]. Another study showed that circulating miR-375 could be used as a biomarker of death in PBCS and diabetes in mice [11]. Currently, there is limited knowledge regarding the role of miRNAs in the regulation of ferroptosis in PBCs.

Hispidin is a polyphenol compound that can be isolated from *Phellinus linteus,* which belongs to the genus *Phellinus* that has been used as a medical mushroom in eastern Asia for a long time [12]. It has been reported that hispidin exerts various biological effects such as anti-inflammatory, antioxidant, and antitumor effects [13]. In addition, hispidin has been reported to have antidiabetic effects [14]. However, there is limited knowledge about the protective effects of hispidin on PBCs against ferroptosis.

In the current study, we investigated the effects of hispidin on high glucose (HG)-treated PBCs and the underlying molecular mechanisms.

## 2. Materials and Methods

### 2.1. Cell Culture and Chemicals

The mouse pancreatic *β*-cell line Min6 was purchased from Thermo Fisher Scientific (USA). Cells were cultured in RPMI 1640 medium (Gibco, USA) supplemented with 10% foetal bovine serum (FBS, Gibco) and 1% penicillin-streptomycin (Beyotime, China) in a humidified atmosphere with 5% CO_2_ at 37°C. Cells were cultured to 70%–80% confluence and treated with glucose (10 mM) for 24 h. Following this, the cells were treated with hispidin for an additional 24 h. Hispidin was purchased from Selleck Chemicals (USA), dissolved in DMSO at a concentration of 1 mM, and stored at −20°C. All other routine chemicals were obtained from Sigma-Aldrich (USA).

### 2.2. Cell Viability Assay

Cell viability assay was performed by using the cell counting kit-8 (Beyotime, China) according to the manufacturer's protocol.

### 2.3. Measurement of Cell Death

Cell death was measured by using the nucleosome ELISA kit (Roche, Switzerland) according to the manufacturer's instructions. The enrichment factor indicated the number of DNA fragments in the cell supernatant or cytoplasm, suggesting cell death. The absorbance was measured on an ELISA reader at a test wavelength of 450 nm. The nucleosome unit value for the unknown sample was calculated by comparing the absorbance of the unknown sample with that of standards.

### 2.4. Cell Transfection

The miR-15b-5p/NC mimic, miR-15b-5p/NC inhibitor, siRNAs against glutaminase (GLS2), si-NC, pcDNA3.1 expressing GLS2 (TFR OV), and pcDNA3.1 (EV) were purchased from RioBio Technology (China). Transfection was performed using Lipofectamine 2000 (Life Technologies, USA) according to the manufacturer's guidelines. The transfection efficiency was assessed by RT-PCR or Western blot.

### 2.5. RT-PCR

The total number of RNA was isolated from the cells by using TRIzol (Life Technologies, USA) according to the manufacturer's instructions. The quality and concentration of RNA were evaluated by using NanoDrop (Thermo Scientific, USA). The miRNAs were reversely transcribed into cDNA by using the miScript transcription kit (Qiagen, Germany) according to the manufacturer's instructions. For the measurement of mRNA, cDNA was synthesized by using the PrimeScript RT reagent kit (Takara, China) according to the manufacturer's guidelines. The expressions of miR-15b-5p and GLS2 were quantified by using the SYBR Green PCR Master Mix Kit (Bio-Rad Laboratories, USA). The relative levels of miR-15b-5p and GLS2 were normalized to U6 and GAPDH, respectively. The calculation was carried out using the 2^−ΔΔCq^ method, and the experiments were repeated three times.

### 2.6. Measurement of Fe^2+^, MDA, ROS, and GSH Levels

The levels of Fe^2+^, MDA, ROS, and GSH were measured by using the iron assay kit, lipid peroxidation (MDA) assay kit, cellular ROS assay kit, and GSH assay kit, respectively, according to the manufacturer's instructions. All the above kits were obtained from Abcam (Cambridge, USA).

### 2.7. Dual-Luciferase Activity Assay

The cells were seeded in 24-well plates at a density of 5 × 10^4^ cells/well. The reporter construct containing the wildtype or mutant GLS2 3′ UTR was cotransfected into the cells with miR-15b-5p using the Lipofectamine 2000 reagent according to the manufacturer's guidelines. The cells were collected 48 h after the transfection, and luciferase activity was measured using the dual-luciferase assay system (Promega, USA).

### 2.8. Microarray Analysis

The miRNA expression analysis was performed based on the Aksomics Technology (China) using the Agilent microarray-based miRNA platform.

### 2.9. Western Blot

Cells were lysed using the RIPA lysis buffer (Beyotime, China). Total proteins (20 *μ*g) were subjected to 12% SDS-PAGE and transferred onto a PVDF membrane (BD Biosciences). The membrane was blocked with 5% skimmed milk for 1 h at room temperature and incubated with primary antibody (Cellular Signalling Technologies, USA) overnight at 4°C. Following this, the membrane was washed thrice with PBS and incubated with the corresponding secondary antibody for 1 h at room temperature. The results were visualized using an ECL reagent (Beyotime, China).

### 2.10. Statistical Analysis

Data are presented as the mean ± standard deviation (SD) from three independent repeated experiments. Statistical analyses were performed using SPSS 11.0 statistical software (IBM, USA). The difference between the two groups was compared using Student's *t*-test. One-way ANOVA followed by Tukey's post hoc test was performed to measure the difference among multiple groups.*P* value of <0.05 indicated a statistically significant result.

## 3. Results

### 3.1. HG Induced Ferroptosis in PBCs

**Figure 1:**
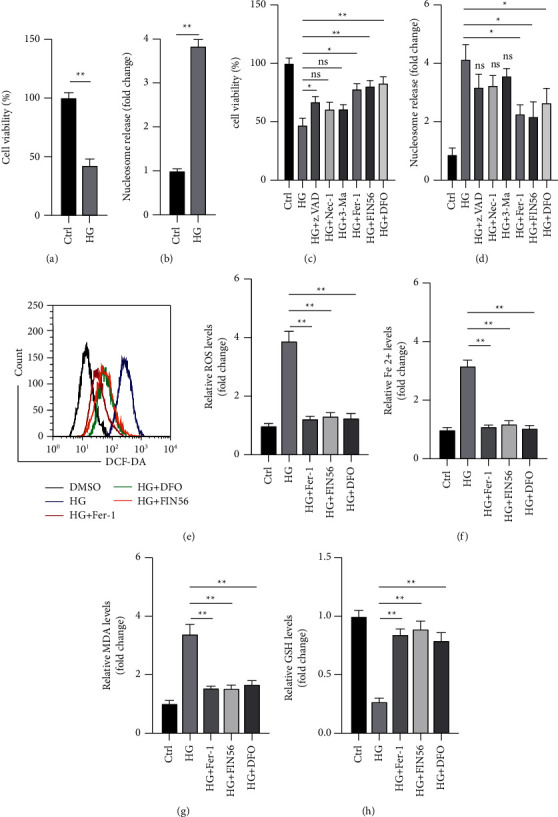
HG treatment caused ferroptosis in PBCs. (a) PBCs treated with glucose (25 mM, HG; 5 mM, ctrl) for 24 h; cell viability was measured. (b) PBCs treated as indicated for 24 h; cell death was measured. (c) PBCs treated with HG alone or with various inhibitors for 24 h; cell viability was measured. (d) PBCs treated with HG alone or with various inhibitors for 24 h; cell death was measured. (e)–(h) PBCs treated with HG alone or with different ferroptosis inhibitors for 24 h. PBCs cultured under normal conditions were used as the control. (e) ROS levels measured. (f) Fe^2+^ levels measured. (g) MDA levels measured. (h) GSH levels measured. Data are representative of at least three independent experiments (^*∗*^*P* < 0.05; ^*∗∗*^*P* < 0.01).

Based on the method described in a previous study, PBCs were treated with 25 mM glucose (HG), which is consistent with the microenvironment of diabetes, and the control cells were treated with 5 mM glucose [15]. The cell viability assay showed that HG treatment reduced the viability of PBCs (Figure 1(a)). Meanwhile, the cell death assay indicated that HG levels induced the death of PBCs (Figure 1(b)). Different inhibitors were applied to identify the type of cell death caused by HG levels. The caspase inhibitor z.VAD was found to marginally promote the viability of PBCs under HG treatment, whereas the necrosis inhibitor Nec-1 and autophagy inhibitor 3-Ma had no such effects (Figure 1(c)). Furthermore, the ferroptosis inhibitors Fer-1 and FIN56 and the iron chelator DFO significantly enhanced the viability of PBCs under the HG condition (Figure 1(c)). The cell death assay showed that z.VAD, Nec-1, and 3-Ma failed to protect PBCs from HG-induced death (Figure 1(d)). Meanwhile, Fer-1, FIN56, and DFO significantly inhibited cell death triggered by HG levels (Figure 1(d)). These data suggested that HG treatment might induce ferroptosis in PBCs. The levels of ROS, Fe^2+^, MDA, and GSH were measured to further confirm that HG treatment caused ferroptosis in PBCs. It was found that HG treatment resulted in the upregulation of ROS, Fe^2+^, and MDA and downregulation of GSH in PBCs (Figures 1(e)–1(h)). In addition, it was found that Fer-1, FIN56, and DFO treatment could abrogate the effects of HG on the changes in ROS, Fe^2+^, MDA, and GSH levels in PBCs (Figures 1(e)–1(h)). Altogether, the findings suggested that HG treatment causes ferroptosis in PBCs.

### 3.2. Hispidin Inhibited the Ferroptosis Caused by HG in PBCs

**Figure 2:**
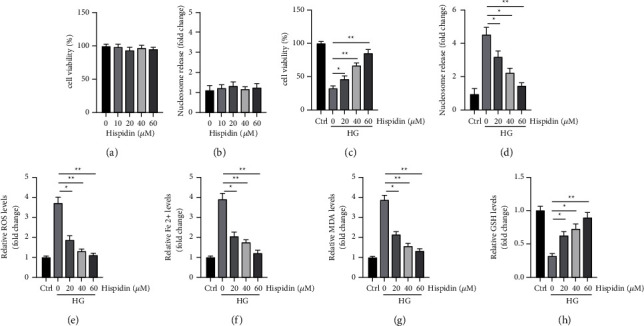
Hispidin inhibits ferroptosis induced by HG levels in PBCs. (a) PBCs treated with various doses of hispidin for 24 h; cell viability was measured. (b) PBCs treated with various doses of hispidin for 24 h; cell death was measured. (c)–(h) PBCs treated with various doses of hispidin under the HG condition for 24 h. PBC cultured under normal conditions was used as the control. (c) Cell viability measured. (d) Cell death measured. (e) ROS levels measured. (f) Fe^2+^ levels measured. (g) MDA levels measured. (h) GSH levels measured. Data are representative of at least three independent experiments (^*∗*^*P* < 0.05; ^*∗∗*^*P* < 0.01).

We investigated whether hispidin protected PBCs from HG levels. PBCs were treated with various doses of hispidin (10, 20, 40, and 60 *μ*M) for 24 h to evaluate the cytotoxicity of the agent. Assessment of cell viability and cell death showed that hispidin was mildly toxic to PBCs (Figures 2(a) and 2(b)). Thereafter, the cells were treated with various doses of hispidin (20, 40, and 60 *μ*M) under the HG condition for 24 h. The cell viability assay showed that hispidin increased viability in a dose-dependent manner under the HG condition (Figure 2(c)). The cell death assay showed that hispidin decreased cell death caused by HG levels in a dose-dependent manner (Figure 2(d)). Furthermore, we investigated whether hispidin has any effects on ferroptosis caused by HG levels in PBC. Interestingly, it was found that hispidin treatment inhibited the upregulation of ROS, Fe^2+^, and MDA caused by HG levels in a dose-dependent manner (Figures 2(e)–2(g)). Furthermore, it was observed that hispidin increased the levels of GSH in a dose-dependent manner under the HG condition (Figure 2(h)). Altogether, the data suggested that hispidin is relatively safe and can protect PBCs against ferroptosis caused by HG levels.

### 3.3. Hispidin Treatment Led to Upregulation of miR-15b-5p in PBCs

**Figure 3:**
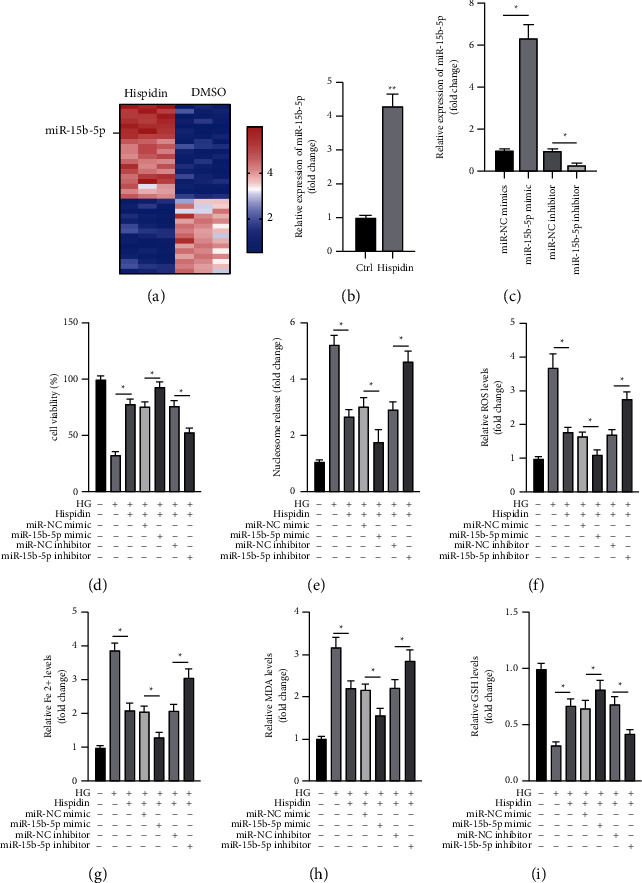
Hispidin treatment led to the upregulation of miR-15b-5p in PBCs. (a) PBCs treated with hispidin (40 *μ*M) or DMSO for 24 h; the expression of miRNAs was assayed using a microarray technique. (b) PBCs treated with hispidin (40 *μ*M) for 24 h; the expression of miR-15b-5p was analyzed by RT-PCR. PBCs treated with DMSO were used as the control. (c) PBCs transfected with the indicated miRNA mimic/inhibitor for 24 h; the expression of miR-802 was and analyzed by RT-PCR. (d)–(i) Under the HG condition, PBCs transfected with the indicated miRNA mimic/inhibitor for 24 h following which the cells were treated with hispidin (40 *μ*M) for an additional 24 h. Cells cultured under normal conditions were used as the control. (d) Cell viability measured. (e) Cell death measured. (f) ROS levels measured. (g) Fe^2+^ levels measured. (h) MDA levels measured. (i) GSH levels measured. Data are representative of at least three independent experiments (^*∗*^*P* < 0.05).

We investigated miRNAs that might be involved in the protective effects of hispidin against HG levels. The microarray results showed that miR-15b-5p was the most upregulated miRNA in PBCs after exposure to hispidin (Figure 3(a)). RT-PCR results also confirmed that hispidin treatment led to the upregulation of miR-15b-5p in PBCs (Figure 3(b)). To evaluate the role of miR-15b-5p in the protective effects of hispidin, we transfected PBCs with a miR-15b-5p mimic or inhibitor to increase or decrease the levels of miR-15b-5p, respectively (Figure 3(c)). The cell viability and cell death assays showed that miR-15b-5p mimic enhanced and the inhibitor reduced the protective effects of hispidin against HG levels in PBCs (Figures 3(d) and 3(e)). Furthermore, it was observed that the effects of hispidin on the changes in ROS, Fe^2+^, MDA, and GSH levels under the HG condition could be enhanced by the miR-15b-5p mimic and be attenuated by the miR-15b-5p inhibitor in PBCs (Figures 3(f)–3(i)). Altogether, the findings suggested that hispidin treatment led to the upregulation of miR-15b-5p, which is an essential for the protective effects of hispidin against ferroptosis caused by HG levels in PBCs.

### 3.4. MiR-15b-5p Negatively Regulates the Expression of GLS2 in PBCs

**Figure 4:**
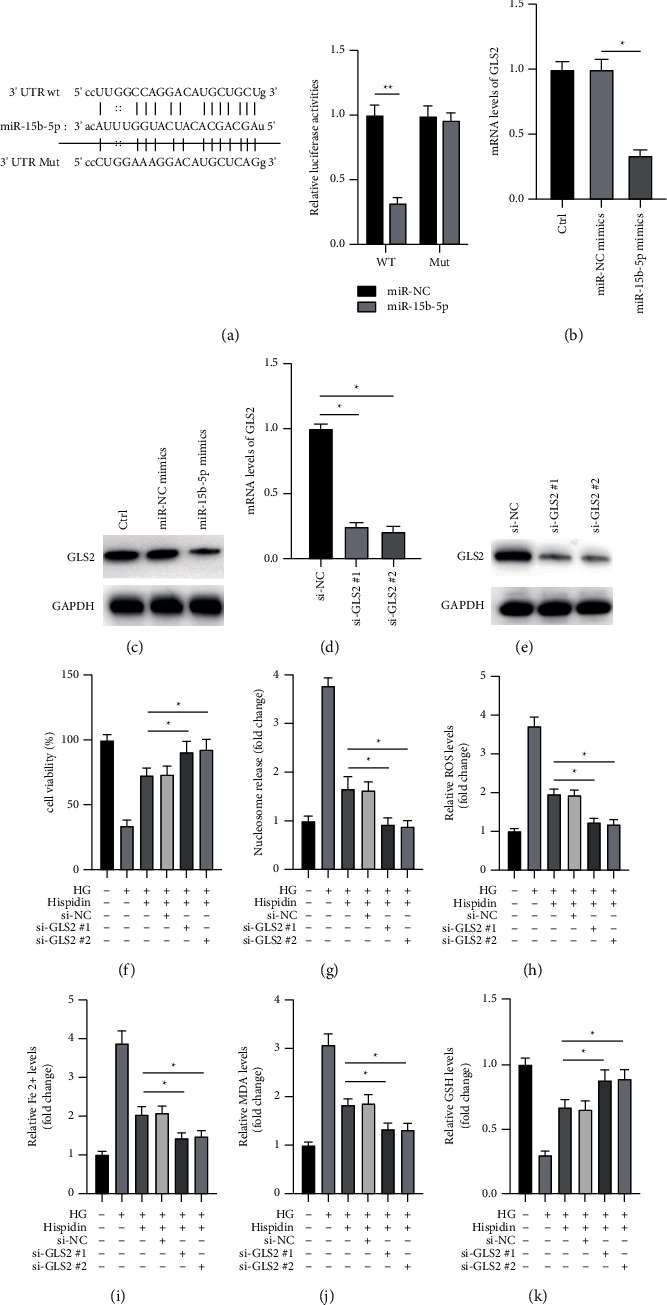
MiR-15b-5p directly targets GLS2 to inhibit ferroptosis induced by HG levels in PBCs. (a) The binding sites between the 3′ UTR of GLS2 and miR-15b-5p have been illustrated (left), and the interaction between miR-15b-5p and the 3′ UTR of GLS2 was verified by the dual-luciferase activity assay. (b) PBCs transfected with miR-NC or miR-15b-5p mimic for 24 h; the mRNA levels of GLS2 were measured by RT-PCR. The untreated PBCS was used as the control. (c) PBCs transfected with miR-NC or miR-15b-5p mimic for 24 h; the protein levels of GLS2 were measured by Western blot. The untreated PBCs were used as the control. (d) PBCs transfected with siRNAs against GLS2 for 24 h; the mRNA levels of GLS2 were measured by RT-PCR. (e) PBCs transfected with siRNAs against GLS2 for 24 h; protein levels of GLS2 were measured by Western blot. (f)–(k) Under the HG condition, PBCs were transfected with different siRNAs for 24 h following which the cells were treated with hispidin for an additional 24 h. Cells cultured under normal conditions were used as the control. (f) Cell viability measured. (g) Cell death measured. (h) ROS levels were measured. (i) Fe^2+^ levels were measured. (j) MDA levels measured. (k) GSH levels measured. Data are representative of at least three independent experiments (^*∗*^*P* < 0.05).

We attempted to identify the downstream target of miR-15b-5p. GLS2 was identified as a potential target of miR-15b-5p by online bioinformatical analysis (starBase 3.0, TargetScan) and gained our attention due to its role in the process of ferroptosis. The dual-luciferase activity assay was performed on PBCs to verify the interaction between miR-15b-5p and GLS2 mRNA. The potential binding sites between miR-15b-5p and 3′ UTR of GLS2 are shown in Figure 4(a). The dual-luciferase activity assay showed that miR-15b-5p significantly inhibited the activity of luciferase in the GLS2 3′ UTR wt group other than the mut group (Figure 4(a)). PBCs were transfected with the miR-15b-5p mimic for 24 h to further confirm that miR-15b-5p can negatively regulate the expression of GLS2. RT-PCR and Western blot assays revealed that the miR-15b-5p mimic decreased the mRNA and protein levels of GLS2 (Figures 4(b) and 4(c)). Furthermore, we attempted to unveil the possible role of the miR-15b-5p/GLS2 axis in the protective effects of hispidin on PBCs against HG levels. We used two siRNAs (si-GLS2 ^#^1 and si-GLS2 ^#^2) to knock down GLS2 in PBCs (Figures 4(d) and 4(e)). The cell viability and cell death assays showed that silencing GLS2 enhanced the protective effects of hispidin on PBCs against HG levels (Figures 4(f) and 4(g)). Measurement of ROS, Fe^2+^, MDA, and GSH showed that knockdown of GLS2 markedly enhanced the protective effects of hispidin on PBCs against ferroptosis caused by HG levels (Figures 4(h)–4(k)). Altogether, the data suggested that the protective effects of hispidin against ferroptosis caused by HG levels might be via the miR-15b-5p/GLS2 axis in PBCs.

### 3.5. Overexpression of GLS2 Attenuated the Protective Effects of Hispidin against HG Levels in PBCs

**Figure 5:**
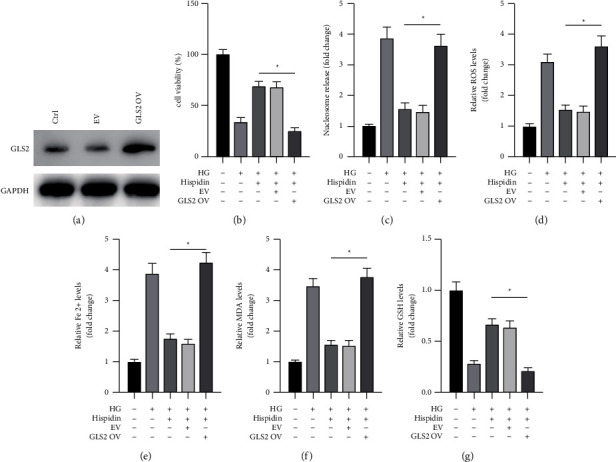
Overexpression of GLS2 abrogates the protective effects of hispidin against ferroptosis induced by HG levels in PBCs. (a) PBCs transfected with an empty vector (EV) or GLS2-overexpressing vector (GLS2 OV) for 24 h; protein levels of GLS2 were measured by Western blot. (b)–(g) Under the HG condition, PBCs were transfected with EV or GLS2 OV for 24 h following which the cells were treated with hispidin for an additional 24 h. Cells cultured under normal conditions were used as the control. (b)Cell viability measured. (c)Cell death measured. (d) ROS levels measured. (e) Fe^2+^ levels measured. (f) MDA levels measured. (g) GSH levels measured. Data are representative of at least three independent experiments (^*∗*^*P* < 0.05).

We transfected PBCs with a vector that overexpressed GLS2 (OV GLS2) to further analyze the role of GLS2 in the protective effects of hispidin against HG levels. It was found that transfection with OV GLS2 significantly increased the protein levels of GLS2 in PBCs compared with the empty vector (EV) (Figure 5(a)). Interestingly, the cell viability and cell death assays showed that the forced expression of GLS2 significantly attenuated the protective effects of hispidin against HG levels in PBCs (Figures 5(b) and 5(c)). Moreover, the overexpression of GLS2 abrogated the effects of hispidin on the changes in ROS, Fe^2+^, MDA, and GSH levels under the HG condition (Figures 5(d)–5(g)). Altogether, the findings suggested that upregulation of GLS2 abrogated the protective effects of hispidin against HG levels by promoting ferroptosis in PBCs.

## 4. Discussion

The death of PBC caused by glucotoxicity is considered a major pathological cause of T2DM [16]. In the current study, we demonstrated that hispidin, a polyphenol, could protect PBCs from damage caused by HG levels. Investigation of the mechanism revealed that hispidin prevented ferroptosis of PBCs caused by HG levels via the miR-15b-5p/GLS2 axis. Our findings provided novel insights into the strategy to prevent the death of PBCs.

Our study showed that a high-glucose medium inhibited the viability and increased the death of PBCs, which is in line with previous studies [17, 18]. We revealed that HG conditions triggered the death of PBCs mainly via ferroptosis. Ferroptosis of PBCs has also been observed in a T2DM mouse model established by streptozotocin injection [19]. Another study revealed that HG levels also caused ferroptosis of osteoblasts, thereby contributing to the pathogenesis of type 2 diabetic osteoporosis [20]. All these findings suggested that ferroptosis plays an essential role in the development of T2DM, and targeting ferroptosis might be a promising strategy.

Hispidin has been found to exert various biological effects including an antidiabetic effect. For example, a study reported that hispidin could overcome palmitate-induced insulin resistance in skeletal muscle myotubes [21]. Another study showed that hispidin could protect PBCs against H_2_O_2_-induced damage [22]. In our study, the cell viability and cell death assays revealed that hispidin did not have any obvious toxic effect on PBCs. Previous studies have also reported that hispidin is nontoxic to retinal pigment epithelial cells, cardiomyoblasts, and adipocytes [23–25]. Therefore, hispidin might be considered a relatively safe agent; however, more in vivo studies are required for further verification. The effects of hispidin on ferroptosis have not yet been reported; however, several studies have revealed that it possesses strong antioxidant activities. For example, hispidin could alleviate H_2_O_2_-induced oxidative stress in PBCs [26]. Hispidin also protects against acrylamide-induced oxidative stress in colon cancer cells [27]. Considering that oxidative stress is critical for the progression of ferroptosis, the protective effects of hispidin against ferroptosis caused by HG levels may depend on its antioxidant property. Furthermore, hispidin has been reported to induce ROS and oxidative stress in colon cancer cells [28]. This discrepancy might be caused by different cell types, and more investigations are required to verify this observation.

To date, many miRNAs are involved in the injury to PBCs caused by HG levels. For example, miR-383 could ameliorate HG-induced apoptosis of PBCs and hyperglycaemia in diabetic mice [29]. MiR-433 was found to protect PBCs against HG-induced damage via negative regulation of COX2 [30]. In the present study, we observed that upregulation of miR-15b-5p was critical for the protective effects of hispidin on PBCs against injury caused by HG levels. Our findings suggested that miR-15b-5p has protective effects against the damage caused by diabetes, which is in line with previous studies. A study reported that HG-induced apoptosis of human kidney cells could be alleviated by overexpression of miR-15b-5p [31]. Another study revealed that miR-15b-5p could ameliorate HG-induced injury of podocytes by inhibiting oxidative stress, apoptosis, and inflammation [32]. However, another study reported that upregulation of miR-15b-5p contributed to the apoptosis of mesangial cells caused by HG levels, thereby promoting diabetic nephropathy [33]. These findings reveal the complex roles of miR-15b-5p in pathogenesis of T2DM. The discrepancy might be caused by different tissues/cells with differential expression of miR-15b-5p. Therefore, more investigations are required to further analyze the role of miR-15b-5p in T2DM.

GLS2 is an essential enzyme for the conversion of glutamine to glutamate and acts as a critical regulator of glutathione (GSH) synthesis and energy generation [34]. PBCs consume a large amount of glutamine and use it for vital biochemical processes such as the synthesis of pyrimidines, purines, and proteins [35]. Deprivation of glutamine has been reported to cause metabolic adaptations along with impairment of the function of PBCs [36]. Therefore, inhibition of GLS2 might maintain or increase glutamine levels, thereby protecting the PBCs. Interestingly, a recent study subjected GLS2 to miR-190-5p regulation and reported that it was involved in the response of cardiomyocytes to ferroptosis [37]. Considering that cardiovascular disease (CVD) is the leading cause of mortality in patients with T2DM, targeting GLS2 might be a potential strategy to treat T2DM, which should be evaluated in future studies.

There are some limitations of our study. First, the bioinformatical analysis showed that hispidin treatment led to changes in various miRNAs. However, we did not investigate whether other miRNAs are involved in the protective effects of hispidin. Second, other signalling pathways might also be involved in the protective effects of hispidin. Third, the effects of hispidin were only evaluated in in vitro, and it would be interesting to test the same in a mouse model in the future.

## 5. Conclusion

In conclusion, we demonstrated that hispidin protected PBCs from HG-induced damage via inhibition of ferroptosis. In addition, hispidin treatment led to the upregulation of miR-15b-5p, which negatively regulates the expression of GLS2 in PBCs. Our findings provide novel insights into the antidiabetic effects of hispidin.

## Data Availability

The data used to support this study are available from the corresponding author upon request.
